# *PHACTR1* modulates vascular compliance but not endothelial function: a translational study

**DOI:** 10.1093/cvr/cvac092

**Published:** 2022-06-02

**Authors:** Alice Wood, Alexios Antonopoulos, Surawee Chuaiphichai, Theodosios Kyriakou, Rebeca Diaz, Abtehale Al Hussaini, Anna-Marie Marsh, Manjit Sian, Mitul Meisuria, Gerry McCann, Victoria S Rashbrook, Edward Drydale, Sally Draycott, Murray David Polkinghorne, Ioannis Akoumianakis, Charalambos Antoniades, Hugh Watkins, Keith M Channon, David Adlam, Gillian Douglas

**Affiliations:** Department of Cardiovascular Sciences, Glenfield Hospital, Leicester, UK; National Institute for Health Research (NIHR) Leicester Biomedical Research Centre, Glenfield Hospital, Leicester, UK; BHF Centre of Research Excellence, Division of Cardiovascular Medicine, Radcliffe Department of Medicine, John Radcliffe Hospital, University of Oxford, Oxford OX3 9DU, UK; BHF Centre of Research Excellence, Division of Cardiovascular Medicine, Radcliffe Department of Medicine, John Radcliffe Hospital, University of Oxford, Oxford OX3 9DU, UK; Wellcome Trust Centre for Human Genetics, University of Oxford, Roosevelt Drive, Oxford, UK; BHF Centre of Research Excellence, Division of Cardiovascular Medicine, Radcliffe Department of Medicine, John Radcliffe Hospital, University of Oxford, Oxford OX3 9DU, UK; Wellcome Trust Centre for Human Genetics, University of Oxford, Roosevelt Drive, Oxford, UK; BHF Centre of Research Excellence, Division of Cardiovascular Medicine, Radcliffe Department of Medicine, John Radcliffe Hospital, University of Oxford, Oxford OX3 9DU, UK; Wellcome Trust Centre for Human Genetics, University of Oxford, Roosevelt Drive, Oxford, UK; Department of Cardiovascular Sciences, Glenfield Hospital, Leicester, UK; National Institute for Health Research (NIHR) Leicester Biomedical Research Centre, Glenfield Hospital, Leicester, UK; Department of Cardiovascular Sciences, Glenfield Hospital, Leicester, UK; National Institute for Health Research (NIHR) Leicester Biomedical Research Centre, Glenfield Hospital, Leicester, UK; Department of Cardiovascular Sciences, Glenfield Hospital, Leicester, UK; National Institute for Health Research (NIHR) Leicester Biomedical Research Centre, Glenfield Hospital, Leicester, UK; Department of Cardiovascular Sciences, Glenfield Hospital, Leicester, UK; National Institute for Health Research (NIHR) Leicester Biomedical Research Centre, Glenfield Hospital, Leicester, UK; Department of Cardiovascular Sciences, Glenfield Hospital, Leicester, UK; National Institute for Health Research (NIHR) Leicester Biomedical Research Centre, Glenfield Hospital, Leicester, UK; BHF Centre of Research Excellence, Division of Cardiovascular Medicine, Radcliffe Department of Medicine, John Radcliffe Hospital, University of Oxford, Oxford OX3 9DU, UK; Wellcome Trust Centre for Human Genetics, University of Oxford, Roosevelt Drive, Oxford, UK; BHF Centre of Research Excellence, Division of Cardiovascular Medicine, Radcliffe Department of Medicine, John Radcliffe Hospital, University of Oxford, Oxford OX3 9DU, UK; Wellcome Trust Centre for Human Genetics, University of Oxford, Roosevelt Drive, Oxford, UK; BHF Centre of Research Excellence, Division of Cardiovascular Medicine, Radcliffe Department of Medicine, John Radcliffe Hospital, University of Oxford, Oxford OX3 9DU, UK; Wellcome Trust Centre for Human Genetics, University of Oxford, Roosevelt Drive, Oxford, UK; BHF Centre of Research Excellence, Division of Cardiovascular Medicine, Radcliffe Department of Medicine, John Radcliffe Hospital, University of Oxford, Oxford OX3 9DU, UK; BHF Centre of Research Excellence, Division of Cardiovascular Medicine, Radcliffe Department of Medicine, John Radcliffe Hospital, University of Oxford, Oxford OX3 9DU, UK; BHF Centre of Research Excellence, Division of Cardiovascular Medicine, Radcliffe Department of Medicine, John Radcliffe Hospital, University of Oxford, Oxford OX3 9DU, UK; BHF Centre of Research Excellence, Division of Cardiovascular Medicine, Radcliffe Department of Medicine, John Radcliffe Hospital, University of Oxford, Oxford OX3 9DU, UK; Wellcome Trust Centre for Human Genetics, University of Oxford, Roosevelt Drive, Oxford, UK; BHF Centre of Research Excellence, Division of Cardiovascular Medicine, Radcliffe Department of Medicine, John Radcliffe Hospital, University of Oxford, Oxford OX3 9DU, UK; Wellcome Trust Centre for Human Genetics, University of Oxford, Roosevelt Drive, Oxford, UK; Department of Cardiovascular Sciences, Glenfield Hospital, Leicester, UK; National Institute for Health Research (NIHR) Leicester Biomedical Research Centre, Glenfield Hospital, Leicester, UK; BHF Centre of Research Excellence, Division of Cardiovascular Medicine, Radcliffe Department of Medicine, John Radcliffe Hospital, University of Oxford, Oxford OX3 9DU, UK; Wellcome Trust Centre for Human Genetics, University of Oxford, Roosevelt Drive, Oxford, UK

**Keywords:** *PHACTR1*, Compliance, Arteries, Endothelial cells

## Abstract

**Aims:**

The non-coding locus at 6p24 located in Intron 3 of *PHACTR1* has consistently been implicated as a risk allele in myocardial infarction and multiple other vascular diseases. Recent murine studies have identified a role for *Phactr1* in the development of atherosclerosis. However, the role of *PHACTR1* in vascular tone and *in vivo* vascular remodelling has yet to be established. The aim of this study was to investigate the role of *PHACTR1* in vascular function.

**Methods and results:**

Prospectively recruited coronary artery disease (CAD) patients undergoing bypass surgery and retrospectively recruited spontaneous coronary artery dissection (SCAD) patients and matched healthy volunteers were genotyped at the *PHACTR1* rs9349379 locus. We observed a significant association between the *PHACTR1* loci and changes in distensibility in both the ascending aorta (AA = 0.0053 ± 0.0004, AG = 0.0041 ± 0.003, GG = 0.0034 ± 0.0009, *P* < 0.05, *n* = 58, 54, and 7, respectively) and carotid artery (AA = 12.83 ± 0.51, AG = 11.14 ± 0.38, GG = 11.69 ± 0.66, *P* < 0.05, *n* = 70, 65, and 18, respectively). This association was not observed in the descending aorta or in SCAD patients. In contrast, the *PHACTR1* locus was not associated with changes in endothelial cell function with no association between the rs9349379 locus and *in vivo* or *ex vivo* vascular function observed in CAD patients. This finding was confirmed in our murine model where the loss of *Phactr1* on the pro-atherosclerosis *ApoE*^−/−^ background did not alter *ex vivo* vascular function.

**Conclusion:**

In conclusion, we have shown a role for *PHACTR1* in arterial compliance across multiple vascular beds. Our study suggests that *PHACTR1* has a key structural role within the vasculature.

## Graphical Abstract

**Graphical abstract:**
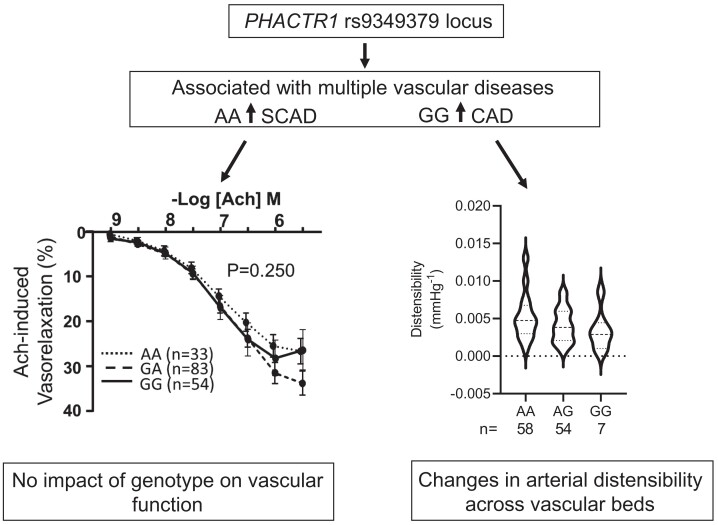

**Time of primary review: 32 days**

## 1. Introduction

Genome-wide association studies have advanced identification of sites of common genetic variation that contribute to increased risk of diseases of medium-sized arteries, including coronary artery disease (CAD). The post-genome-wide association study (GWAS) challenge is to identify the genes that confer the causative association with each risk locus and discover the biological mechanisms linking these genes to disease. Multiple GWASs have independently identified a non-coding locus at 6p24 as being associated with CAD.^1–3^ Fine-mapping studies have identified rs9349379, which sits within the third intron of the gene encoding phosphatase and actin regulatory protein 1 (*PHACTR1*) as the causal CAD-risk variant.^4^ This locus has also been associated with multiple other vascular phenotypes such as coronary microvascular dysfunction,^5^ cervical artery dissection,^6^ spontaneous coronary artery dissection (SCAD),^7^ hypertension,^8^ fibromuscular dysplasia,^9^ and migraine.^10^ The risk allele across diseases is not uniform, for example CAD is associated with the AA allele, whereas SCAD is associated with the GG allele. The association of this locus with multiple vascular diseases strongly implicates this region as being important in vascular function.^11^

The causal gene mediating the biological effects of variation at the 6p24 locus was initially debated, with some studies suggesting a role for endothelin-1.^11^ However, extensive studies have now strongly implicated *PHACTR1* as the causal gene. Decreased expression of *PHACTR1* mRNA with no change in endothelin-1 mRNA was observed in isogenic iPSC-derived endothelial cells carrying the rs9349379 CAD-risk allele.^12^ The rs9349379 CAD-risk allele (GG) was also shown to be associated with reduced *PHACTR1* expression in the aorta, tibia, and coronary artery.^4,12^ In addition, this variant was also shown to alter binding of myocyte enhancer factor-2 (MEF2). Deletion of the MEF2 binding site at this locus was associated with reduced *PHACTR1* expression.^4^

Recent evidence has pointed to a causal role of *PHACTR1* in the development of atherosclerosis via modulation of monocyte/macrophage function. Loss of *Phactr1* globally or specifically in monocytes is associated with an increased atherosclerotic burden.^13,14^ A key role for *Phactr1* has also been demonstrated in endothelial cells where loss of *Phactr1* was associated with reduced angiogenesis, proliferation, and increased apoptosis.^15,16^ To date no study has investigated the *in vivo* role of *Phactr1* in vascular function. In this study, we sought to establish the role of *PHACTR1* in vascular function and *in vivo* vascular remodelling. In patients with either SCAD or CAD.

## 2. Methods

All human studies were ethically approved and conducted in patients with their fully informed consent and in accordance with the Declaration of Helsinki.

### 2.1 Clinical studies on endothelial cell function

Patients undergoing elective cardiac surgery coronary artery bypass grafting (CABG) at the John Radcliffe Hospital, Oxford University Hospitals NHS Trust, were recruited to the Oxford Cohort for Heart, Vessels and Fat (approved by the UK Human Research Authority and the UK National Research Ethics Service study reference MREC 11/SC/0140). Patients with active inflammatory, neoplastic, renal, or hepatic disease were excluded. The demographic characteristics are presented in Supplementary material online, *Table S1*.

Flow-mediated dilatation (FMD) and endothelium-independent vasodilatation (EID) of the brachial artery were measured the day before surgery using a linear array transducer and automated off-line analysis (Vascular Analyser, Medical Imaging Applications LLC). For FMD measurement, brachial artery diameter was recorded before and for a period of 60 s after a 5-min forearm blood flow occlusion. EID was assessed 3 min after a sublingual spray of glyceryl trinitrate (400 µg). FMD and EID of the brachial artery were defined as the % change in vessel diameter from baseline.

Vasomotor studies were performed in saphenous vein segments obtained during CABG, as previously described.^17^ In brief, vessel rings were equilibrated in oxygenated (95% O_2_/5% CO_2_) Krebs–Henseleit buffer (KHB) at 37°C to achieve a resting tension of 3 g. Vessel rings were precontracted with phenylephrine (PE) (3 × 10^−6^ M); then endothelium-dependent relaxations were quantified using acetylcholine (Ach, 10^−9^ to 10^−5.5^ M) and bradykinin (BK, 10^−9^ to 10^−5.5^ M). Relaxations to the endothelium-independent NO donor sodium nitroprusside (SNP, 10^−10^ to 10^−6^ M), were evaluated in the presence of the NOS inhibitor NG-nitro-L-arginine methyl ester (L-NAME; 100 μM).

### 2.2 Clinical studies of arterial distensibility and strain

The UK SCAD registry [approved by the UK National Research Ethics Service (14/EM/0056)] collected data on patients with angiographically confirmed SCAD from across the UK by referral from the clinical team at the presenting hospital, primary care referral, or self-referral to an online web portal. Between 2015 and 2019 patients from the UK SCAD Registry and healthy controls recruited by open advertisement and targeted to match the age/sex profile of the SCAD cohort were invited to participate in the SCAD Deep Phenotyping Study (ISRCTN42661582). The demographic characteristics are presented in Supplementary material online, *Table S2*.

Cardiac magnetic resonance imaging (MRI) was used to establish aortic distensibility, a direct measure of aortic stiffness, in both the ascending and descending aorta. Cardiac MRI has good agreement compared with invasive measurements with excellent reproducibility.^18^ Steady-state free precession aortic cine images were acquired in a plane perpendicular to the thoracic aorta at the level of the pulmonary artery bifurcation as previously described^19,20^ with simultaneous brachial blood pressure measurement. Aortic distensibility was analysed by a single operator blinded to clinical status and genotype using Java Image Manipulation version 6 (Xinapse Software, Essex, UK) blinded to all participants data. Distensibility was calculated as:


Distensibility=(AreaMax–AreaMin)/(Areamin×pulsepressure)

Carotid ultrasound was used to establish carotid strain. Ultrasounds were analysed blinded to clinical status and genotype using Carotid Analyzer for Research version 6.4.8, Medical Imaging Applications Ltd., a semi-automated edge detection system. Images were imported into this system and a region of interest was selected on a portion of the vessel that was clearly visualized. The media-to-media distance was measured. Images analysed by this system were inspected and if tracking was clearly erroneous they were manually amended where possible, or excluded. The maximum and minimum diameters were then used to calculate the percentage change in diameter across the cardiac cycle. This was done for the right and left carotid arteries separately and the mean change across both arteries was also calculated.


%Strain=[(Maxdiameter−MinDiameter)/MinDiameter]

### 2.3 Animals

A targeting vector, HTGRS6013_A_D10, suitable for the generation of a Knock-out first *Phactr1* allele was obtained from the Knock-out Mouse Project^21^ via the Children’s Hospital and Research Centre at Oakland. Following homologous recombination in JM8F6 embryonic stem cells, an FRT flanked IRES-lacZ-pA cassette linked to a strong splice acceptor signal, together with a loxP flanked neomycin selection cassette was integrated into Intron 6 of *Phactr1* (with respect to the ENSMUST00000110161 *Phactr1* transcript) and an additional loxP site was incorporated into Intron 7, thus floxing Exon 7 (ENSMUSE00000493553) of the *Phactr1* gene. Targeted deletion of this exon has previously been shown to alter atherosclerosis burden.^13^ Recombinant ES cells were microinjected into albino C57BL/6 blastocysts and three resulting chimeric offspring with 50–70% chimerism were selected for breeding. Flp-mediated excision of the Splice-Acceptor-LacZ-pA cassette was carried out by breeding the chimeric males with a Flp deleter female [Tg (ACTB-FlpE) 9205Dym/J] on a C57BL/6J background, allowing the generation of a floxed *Phactr1* allele (*Phactr1^fl/fl^*). In order to generate mice globally deficient in *Phactr1*, *Phactr1*^fl/fl^ mice were crossed with Sox2Cre mice [Tg (Sox2-cre) 1Amc/J)]. The progeny of this cross was bred with *ApoE*^−/−^ mice (B6.129P2-Apoetm1Unc/J) to generate mice with heterozygous deletion of *Phactr1* and *ApoE*. Mice were backcrossed with *ApoE*^−/−^ on the C57BL/6J background for >8 generations.

The generation and phenotyping of the knock-out model were carried out in accordance with the Animal (Scientific Procedures) Act 1986, with procedures reviewed by the clinical medicine animal care and ethical review body, and conducted under project licenses PPL 30/3080 and P0C27F69A. Animals were housed in individually ventilated cages (between 4 and 6 mice per cage of mixed genotypes) in specific pathogen-free conditions. All animals were provided with standard chow (B&K Ltd., UK) and water ad libitum and maintained on a 12 h light:12 h dark cycle at controlled temperature (20–22°C) and humidity. Heart rate and systolic blood pressure were measured (between 9 and 11 am) using an automated computerized tail-cuff system in 20–22-week-old male and female mice, as described previously (Visitech BP2000, Visitech Systems Inc., USA).^22^ All mice were culled by exsanguination under terminal anaesthetic (isoflurane >4% in 95%O_2_ 5%CO_2_); depth of anaesthesia was monitored by respiration rate and withdrawal reflexes. Tissue for biochemical analysis was collected from mice perfused with phosphate-buffered saline and snap frozen in liquid nitrogen and stored at −80°C until analysis. Total RNA was extracted using the Ambion Pure Link kit. Quantitative real-time RT-PCR was performed with an iCycler IQ real-time detection system (BioRad Laboratories) using primers and probes from the *Taq*Man Gene Expression Assay system (Life Technologies). Gene expression data were normalized to an appropriate house keeper using the delta CT method.

All animal procedures were approved and carried out in accordance with the University of Oxford ethical committee and the UK Home Office Animals (Scientific Procedures) Act 1986. All procedures conformed to the Directive 2010/63/EU of the European Parliament.

### 2.4 Isometric tension vasomotor studies

Vasomotor function was analysed using isometric tension studies in a wire myograph (Multi-Myograph 610 M, Danish Myo Technology, Denmark). Briefly, adult male mice (16–19 weeks old) were culled by overdose of inhaled isoflurane. The descending aorta was excised from the mouse and placed in cool KHB [(in mmol L^−1^): NaCl 120, KCl 4.7, MgSO_4_ 1.2, KH_2_PO_4_ 1.2, CaCl_2_ 2.5, NaHCO_3_ 25, glucose 5.5]. Segments of aorta were carefully dissected free from surrounding fat and connective tissue as described.^23,24^ The arteries (2 mm) were mounted on a wire myograph containing 5 ml of KHB at 37°C, gassed with 95% O_2_ 5% CO_2_. After allowing vessels to equilibrate for 30 min, aortas were set to an optimal resting tension. The vessel viability was tested using 45 mmol L^−1^ KCl. Concentration–response contraction curves were established using cumulative half-log concentrations to PE. Vessels were washed three times with fresh KHB, equilibrated for 20 min, and then precontracted to ∼80% of maximal tension with PE. Acetylcholine (1−10 μmol L^−1^) was used to stimulate endothelium-dependent vasodilatations in increasing cumulative concentrations. Responses were expressed as a percentage of the precontracted tension. Finally, the NO donor SNP (0.1–1 μmol L^−1^) was used to test endothelium-independent smooth muscle relaxation in the presence of L-NAME. All pharmacological drugs were pre-incubated at least 20 min before the dose–response curves were determined L-NAME was used at 100 µM.

### 2.5 Statistical analysis

Data are presented as mean ± SEM. Normality was tested using the Shapiro–Wilk test. Groups were compared using the Mann–Whitney *U* test for non-parametric data or an un-paired Student’s *t*-test for parametric data. When comparing multiple groups data were analysed by analysis of variance (ANOVA) with Newman–Keuls post-test for parametric data or Kruskal–Wallis test with Dunns post-test for non-parametric data. When more than two independent variables were present a two-way ANOVA with Tukey’s multiple comparisons test was used. When within-subject repeated measurements were present a repeated measures (RM) ANOVA was used. A value of *P* < 0.05 was considered statistically significant. All experiments and analysis were carried out by personnel blinded to genotype. The experimental unit was defined as a single animal, animals of both genotypes were caged together and in all experiments animals of both genotypes were derived from more than one cage. Age- and sex-matched mice were randomly assigned to experiments.

For clinical studies, continuous variables were tested for normal distribution using the Kolmogorov–Smirnov test. Non-normally distributed variables were log-transformed for analysis. Continuous variables were compared by using one-way ANOVA followed by Bonferoni *post hoc* test when individual comparisons were applied.

## 3. Results

### 3.1 *PHACTR1* variants are not associated with altered endothelial function in CAD patients

To test for associations between *PHACTR1 g*enotype and changes in vascular function, we genotyped prospectively recruited patients undergoing elective cardiac surgery for the *PHACTR1* eQTL SNP rs9349379.

**Figure 1:**
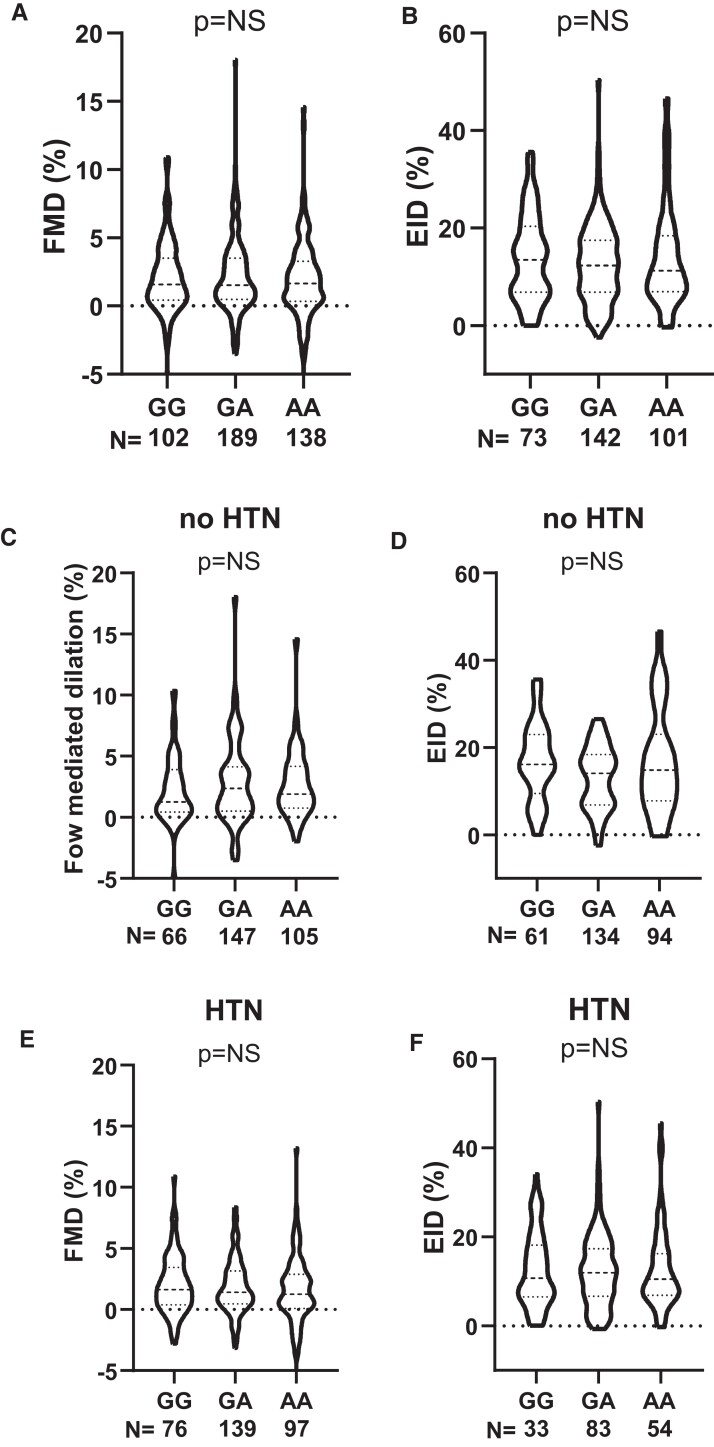
*PHACTR1* coronary artery disease risk allele (GG) did not impact on *in vivo* vascular function. (*A*) *In vivo* dilator response to flow (FMD) was not different across the genotypes (GG; *P* > 0.05, one-way ANOVA, GG = 102, GA = 189, and AA = 138 subjects per genotype). (*B*) No difference between genotypes was observed in endothelial cell-independent dilation (EID, GG = 73, GA = 142, and AA = 101 subjects per genotype) in response to GTN *in vivo*. Population was subdivided into non-hypertensive (no HTN). (*C*) (G = 66, GA = 147, and AA = 105 subjects per genotype) and (*D*) (G = 61, GA = 134, AA = 94 subjects per genotype) and hypertensive (*E*) (G = 76, GA = 139, and AA = 97 subjects per genotype) and (*F*) (G = 33, GA = 83, and AA = 54 subjects per genotype) no difference was observed in either FMD or EID across the genotypes in either population.

In order to test the influence of *PHACTR1* variants on endothelial cell function *in vivo*, we quantified brachial artery flow-mediated vasodilation using ultrasound measurement of brachial artery diameter before and after a brief occlusion of the vessel by suprasystolic inflation of a blood pressure cuff. There was no difference across the genotype in flow-mediated dilation (*Figure 1A*). In addition, the CAD-risk allele did not alter sensitively of the VSMCs to nitric oxide, since endothelial cell-independent dilation in response to GTN was not different between genotypes (*Figure 1B*). We subdivided this cohort into CAD patients into those who had hypertension (defined as a blood pressure >140/90 mmHg) and non-hypertensive. There was no significant difference in either group in either FMD or EID (*Figure 1C–F*).

**Figure 2:**
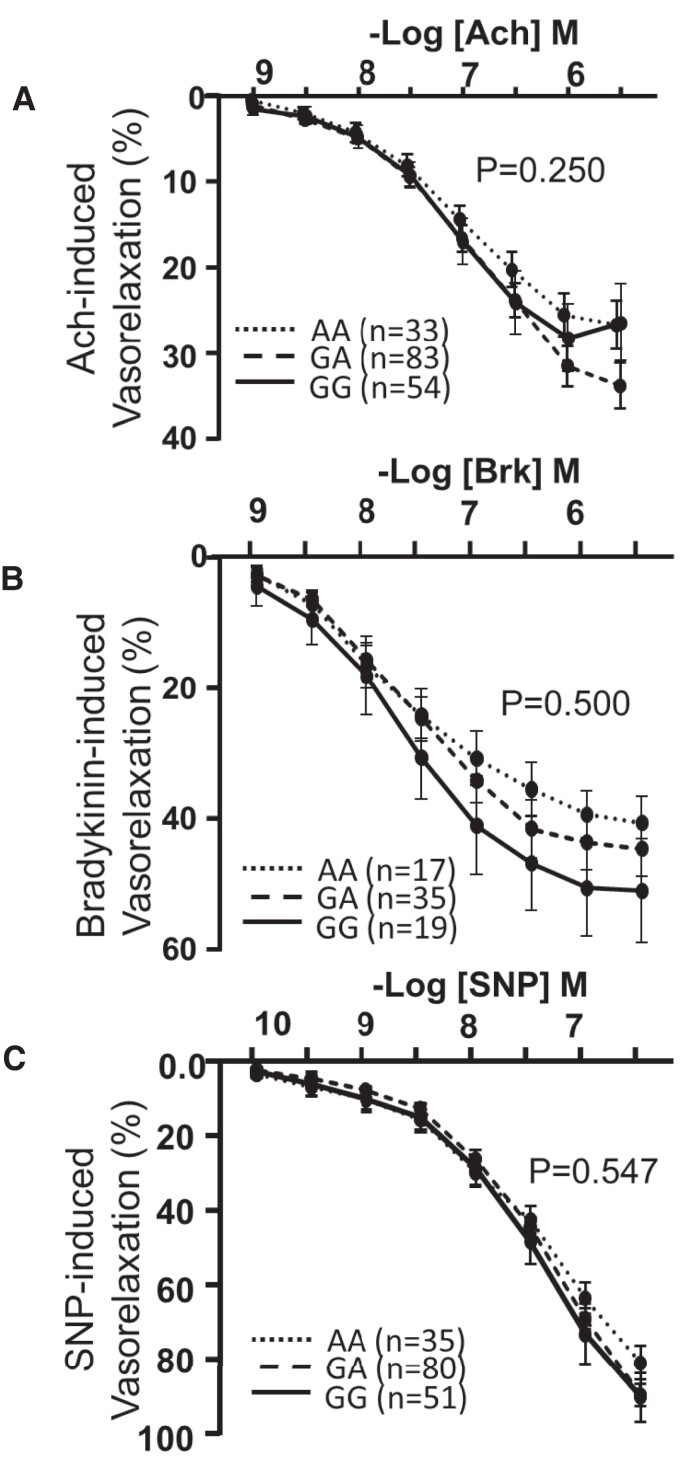
*PHACTR1* coronary artery disease risk allele (GG) did not impact on *ex vivo* vascular function. Endothelial cell-dependent dilation to acetylcholine (*A*) GG = 33, GA = 83, and AA = 54 subjects per genotype and bradykinin (*B*) GG = 17, GA = 35, and AA = 19 subjects per genotype was assessed in saphenous veins there was no difference observed across the genotypes (*P* > 0.05, two-way ANOVA for repeated measures). (*C*) Endothelial cell-independent dilation in saphenous veins to sodium nitroprusside (SNP, GG = 35, GA = 80, and AA = 51 subjects per genotype) was not different between genotypes.

These *in vivo* studies were supported by *ex vivo* organ bath measurements of endothelial cell function in saphenous vein rings harvested at the time of cardiac surgery, revealing no difference in the sensitivity to the endothelial cell-dependent vasodilator BK or acetylcholine or to the endothelium-independent dilator, SNP across the genotypes (*Figure 2A–C*).

### 3.2 *PHACTR1* variants are associated with altered vascular distensibility

**Figure 3:**
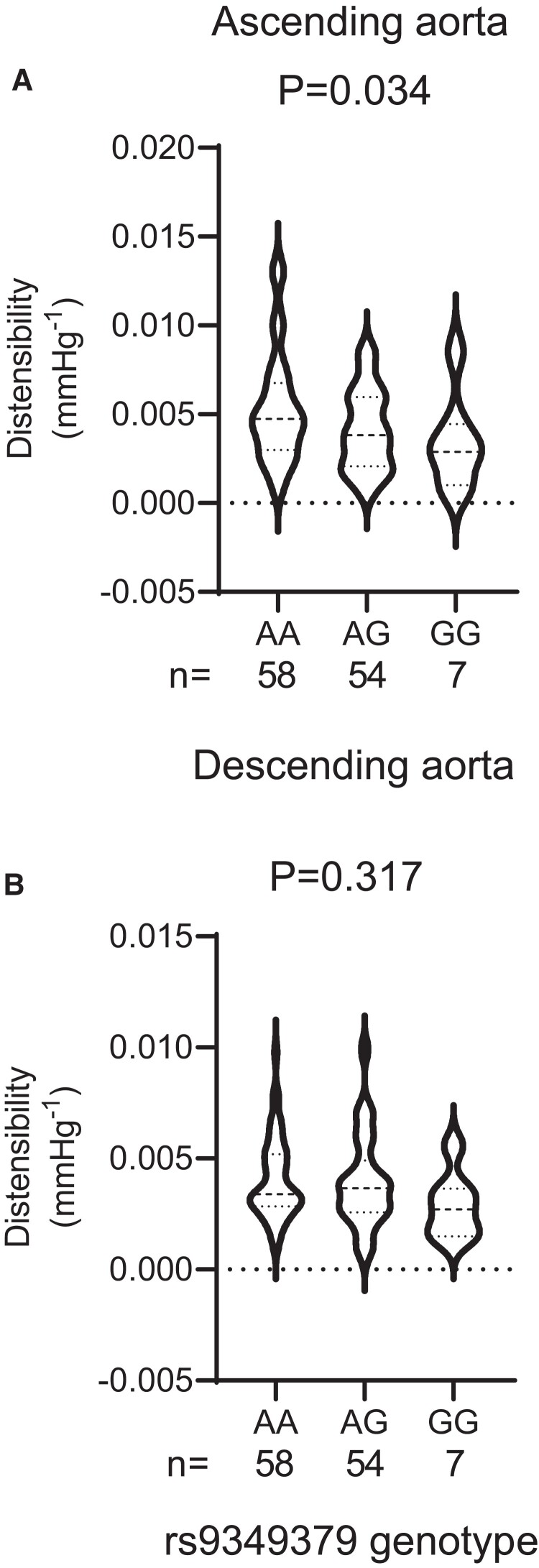
Carriers of the *PHACTR1* coronary artery disease risk allele (GG) had reduced ascending aorta distensibility compared with carriers of the spontaneous coronary artery dissection allele (AA). (*A*) Ascending aorta distensibility was significantly decreased in carriers of the GG allele compared with carriers of the AA allele *P* = 0.034: one-way ANOVA. (*B*) No difference between genotypes was observed in distensibility in the descending aorta (*P* = 0.317: one-way ANOVA, AA = 58, AG = 54, GG = 7).

In order to determine if the *PHACTR1* variant was associated with changes in vascular distensibility we genotyped prospectively recruited SCAD patients and healthy volunteers (HV) for the rs9349379 SNP. SCAD patients and HV were matched for age, sex, and BMI (Supplementary material online, Table S2). As previously reported within this population the AA genotype is associated with the risk of SCAD and increased *PHACTR1* expression. We observed a significant association between the *PHACTR1* loci and ascending aorta distensibility with increased distensibility observed in carriers of the AA allele compared with carriers of the GG allele (*Figure 3A*). This association was not observed in the descending aorta where no significant association between genotype and distensibility was observed (*Figure 3B*).

**Figure 4:**
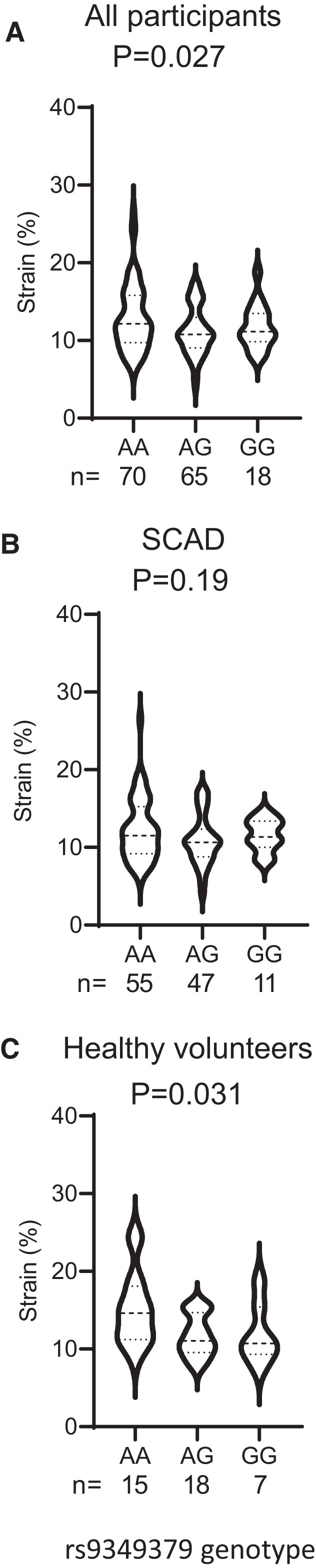
Healthy volunteer carriers of the GG allele but not spontaneous coronary artery dissection (SCAD) patients had a reduction in carotid artery distensibility. (*A*) Carotid artery strain was significantly reduced in carriers of the GG allele in the combined study group (*P* = 0.027, one-way ANOVA, AA = 70, AG = 65, GG = 18). (*B*) In SCAD, patient sub-group no difference in strain was observed with genotype (*P* = 0.19, one-way ANOVA, AA = 55, AG = 47, GG = 11). (*C*) Healthy volunteers showed a significant reduction in strain with genotype with reduced strain observed in carriers of the GG allele (*P* = 0.031, one-way ANOVA, AA = 15, AG = 18, GG = 7).

We next assessed distensibility at a second location, the carotid artery. As with the AA we observed a significant difference in distensibility at the *PHACTR1* loci with an increased distensibility observed in carriers of the AA genotype compared with carriers of the GG genotype (*Figure 4A*). We subdivided this population in the patients who had a SCAD and HV. Interestingly, the reduction in distensibility was driven by differences in the HV population with no significant relationship between distensibility and genotype observed in the SCAD group (*Figure 4B and C*).

### 3.3 Loss of *Phactr1* does not alter blood pressure but does lead to an increase in heart rate

**Figure 5:**
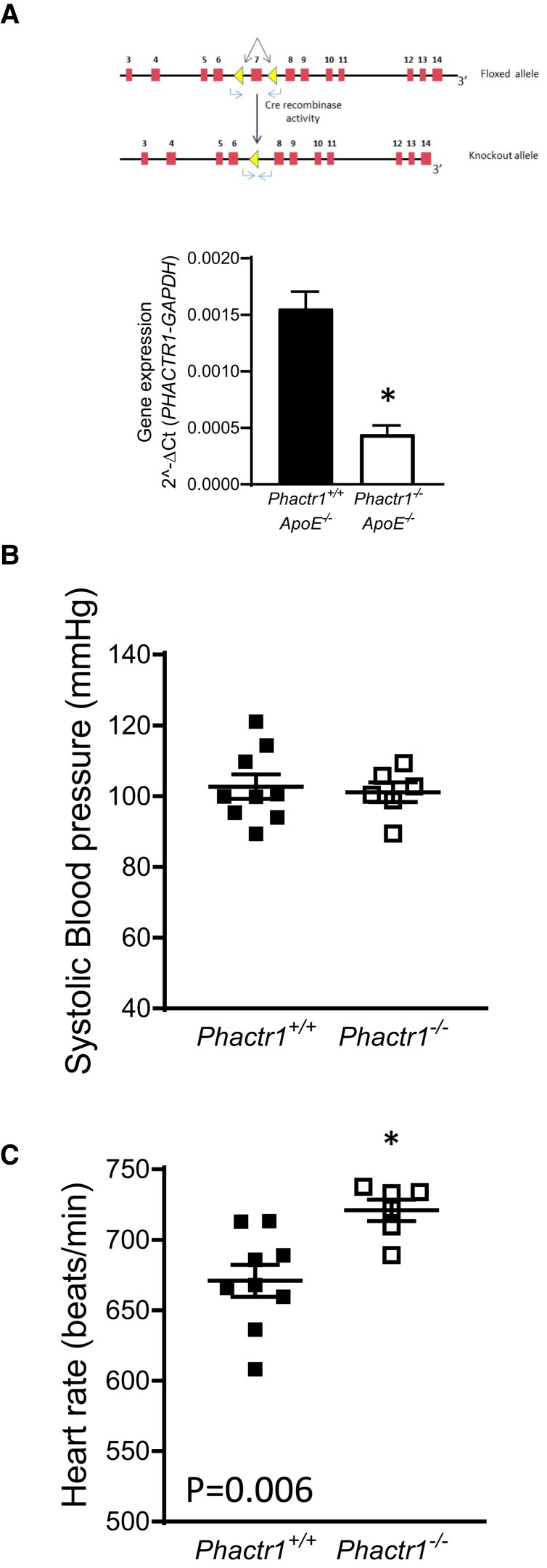
Loss of *Phactr1* causes a significant increase in heart rate. (*A*) Schematic showing the targeting of the murine *Phactr1* locus with loxP sites flanking Exon 7, mRNA analysis showing a significant reduction in *Phactr1* expression in hearts from *Phactr1*^−/−^*ApoE*^−/−^ mice (*P* < 0.05, *T*-Test, adult males, *n* = 4 *Phactr1^+/+^ApoE*^−/−^ and *n* = 5 *Phactr1^+/+^ApoE*^−/−^). (*B*) Systolic blood pressure was not significantly different between groups (*P* > 0.05, *T*-test). (*C*) A significant increase in heart rate was observed in *Phactr1*^−/−^*ApoE*^−/−^ mice compared with their *Phactr1^+/+^ApoE*^−/−^ control littermates (*P* < 0.05, *T*-test). Adult mice between 20 and 22 weeks of age, *n* = 4 female and 5 male *Phactr1^+/+^ApoE*^−/−^ and 3 female and 3 male *Phactr1*^−/−^*ApoE*^−/−^ mice. Data are expressed as the mean ± SEM, each point represents an individual animal. Black bars/symbols = *Phactr1^+/+^*, white bars/symbols = *Phactr1*^−/−^.

In order to investigate the mechanistic role of *Phactr1* in vascular function, we generated global *Phactr1* knock-out (*Phact1*^−/−^) mice. In order to mimic the metabolic dysregulation commonly associated with cardiovascular disease, we crossed these mice onto the hyperlipidaemic *ApoE* knock-out background. PCR analysis of genomic DNA confirmed deletion of Exon 7 in *Phactr1*^−/−^ mice. cDNA from knock-out mice showed the expected reduction in band size when primers spanning Exons 4–13 were used, sequencing of cDNA from knock-out mice confirmed excision of Exon 7 (data not shown). A significant reduction in *Phactr1* expression was observed in heart tissue from *Phact1*^−/−^*ApoE*^−/−^ mice (*Figure 5A*).

We next assessed how loss of *Phactr1* impacted on hemodynamic control. No difference was observed in systolic blood pressure with loss of *Phactr1* (*Figure 5B*). However, loss of *Phactr1* did result in a small but significant increase in heart rate from 670 to 720 beats/min (*Figure 5C*).

### 3.4 Loss of *PHACTR*1 did not alter vascular function

**Figure 6:**
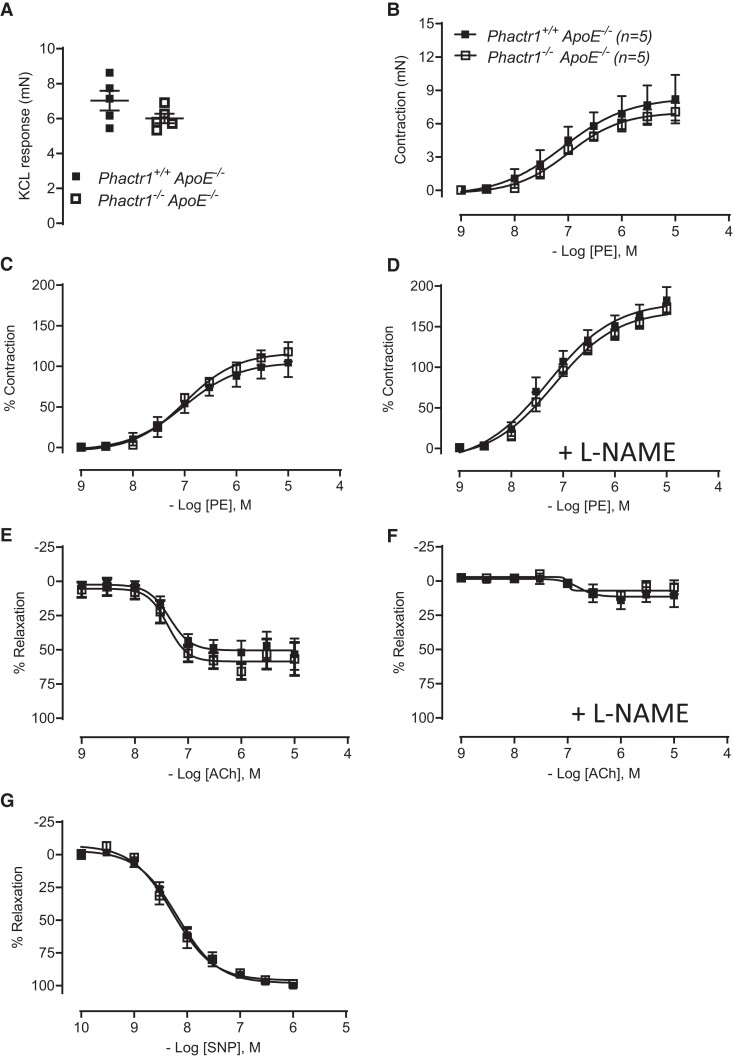
No difference in vasomotor motor function in the aorta of *Phactr1*^−/−^*ApoE*^−/−^ mice. Vasomotor function in the aorta of *Phactr1^+/+^ApoE*^−/−^ and *Phactr1*^−/−^*ApoE*^−/−^ was determined using isometric tension studies in a wire myograph. (*A*) Force of maximal contraction to 45 mmol L^−1^ KCl. Receptor-mediated vasoconstriction to phenylephrine (PE) expressed in absolute tension (*B*) and as % of maximum KCL constriction to control for variation in vessel size (*C*). (*D*) Vasoconstriction to PE in the presence of NOS inhibitor, L-NAME (100 μmol L^−1^). Receptor-mediated endothelium-dependent vasodilatation to ACh in the absence (*E*), presence of L-NAME (*F*), endothelium-independent vasodilatation to SNP (*G*). No significant differences were observed between groups (*P* < 0.05, RM ANOVA); *n* = 5 male adult (16–19 weeks old) mice per group. Black symbols = *Phactr1^+/+^*, white symbols = *Phactr1*^−/−^.

We next aimed to establish if global loss of *Phactr1* altered vasomotor function. To mimic the metabolic dysfunction observed in our clinical population we assessed vasomotor function in *Phactr1*^−/−^ mice on the *ApoE*^−/−^ background. Isometric tension studies in isolated aortas demonstrated that the vasoconstriction response to phenylephrine in both absolute values and when normalized to a maximum constriction dose of KCl was comparable between genotypes (*Figure 6B and C*). As expected the presence of L-NAME lead to an increased constrictor response due to the tonic inhibition of NO production, however, the lack of *Phactr1* did not impact on this response (*Figure 6D*). Endothelial cell-dependent relaxation to acetylcholine was not impacted by the loss of *Phactr1*, this response was almost completely abolished by the presence of L-NAME in both groups indicating that in both genotypes NO mediated this response (*Figure 6E and F*). In addition, no difference was observed in the endothelial cell-independent dilation to SNP between groups (*Figure 6G*).

## 4. Discussion

The *PHACTR1* locus rs9349379 is associated with multiple vascular diseases; however, the vascular phenotype resulting from variation at this locus has yet to be fully established. We have shown that on a pro-atherosclerotic *ApoE*^−/−^ background loss of *Phactr1* in mice did not impact blood pressure or vascular function. These *in vivo and ex vivo* vascular function data in mice were supported by clinical data which showed no association between the *PHACTR1* rs9349379 locus and *in vivo* and *ex vivo* vascular function in a cohort of patients with advanced CAD. However, this locus was associated with changes in arterial distensibility with the SCAD-risk allele associated with increased distensibility compared with the CAD-risk allele in both the ascending aorta and carotid artery.

No studies have investigated the role of *PHACTR1* in vascular function. We show using genetically modified mice that loss of *Phactr1* on a pro-atherogenic *ApoE*^−/−^ background is not associated with changes in endothelial cell-dependent or independent vasodilation nor any difference in contractile function. This finding is in keeping with findings from our clinical studies where no association between the *PHACTR1* locus and *in vivo* and *ex vivo* endothelial function was observed in a clinical population with advanced CAD. A previous study using data from the CHARGE consortium found a significant reduction in flow-mediated dilation in carriers of the GG (rs9349379) allele which is associated with reduced *PHACTR1* expression.^11^ The difference in these two studies may be due to differences in study populations. Our study was carried out in a population with advanced CAD where a small difference in endothelial cell function may no longer be apparent due to the attenuation of FMD arising from arterial disease. It would be interesting to investigate arterial function in HV and knock-out mice on an *ApoE*^+/+^ background to address the question. Interestingly, *in vitro* studies in primary endothelial cells show loss of *PHACTR1* to be anti-atherogenic, with a reduction in inflammatory adhesion molecule expression observed in response to oxidized LDL.^25^ This indicates that the loss of *PHACTR1* in endothelial cells may not lead to a detrimental endothelial cell phenotype in a hyperlipidaemic environment. Taken together these studies do not implicate loss of *PHACTR1* in a detrimental functional endothelial cell phenotype. Loss of *Phactr1* was associated with an increase in heart rate, however, the change in heart rate was not associated with an increase in blood pressure. Further studies are required to elucidate if this increase is due to an indirect or direct action of *Phactr1*. *Phactr1* has been shown to modulate the function of the KCTN channel,^26^ modulation of this channel or other yet unidentified ion channels could be responsible for these changes.

Arterial distensibility is a measure of the arterial ability to expand and contract with cardiac pulsation and relaxation. Decreased distensibility leads to arterial stiffness which is an independent predictor for cardiovascular diseases including CAD.^27^ We show that the CAD allele GG (rs9349379; associated with reduced *PHACTR1* expression) is associated with decreased distensibility in the ascending aorta compared with the SCAD-risk allele AA which is associated with increased distensibility. This finding indicated that the increased CAD-risk associated with the GG allele may be in part mediated by changes in arterial distensibility. The ascending aorta plays a key role in vascular-ventricular coupling with decreased ascending aorta distensibility a significant predictor of all-cause mortality and hard cardiovascular disease endpoints independent of age and traditional risk factors.^28^ Although the ascending aorta is the major contributor to the Windkessel function the descending aorta also plays a key role in this response thus changes in distensibility at this location will also impact cardiovascular disease risk. Interestingly, no significant difference across the genotypes was observed in the descending aorta, potentially indicating a region specific change in compliance across the genotypes. The ascending and descending aorta have different embryonic origins and there are significant differences in elasticity between these regions which become greater with age.^29^ Indeed, previous studies which measure arterial stiffness index by photoplethysmography in upper extremities have shown the CAD-risk allele (GG) to be associated with decreased arterial stiffness.^11^ This is in contrast to the findings in this study where GG was associated with increased arterial stiffness. Measurement of arterial stiffness at different locations likely measures unique location-specific properties. Proteomic studies have shown significant regional difference in protein expression in vascular smooth muscle cells.^30^ The regional difference in distensibility may indicate a differential role of *PHACTR1* at different arterial locations. Arterial stiffness is a complex interplay between endothelial and vascular smooth muscle cell function and extracellular matrix.^30^ Endothelial cells, via release of NO and EDHF, have been shown to have a key role in arterial stiffness.^31^ In this study, we did not show any difference in endothelial cell-dependent vasodilation between wild type and *Phactr1* knock-out mice or *in vivo* and *ex vivo* endothelial cell function in our clinical study. This indicates that the changes in distensibility observed in the current study are not likely mediated by a *PHACTR1*-dependent changes in dynamic vascular function. However, differences in contractile function have been observed between the ascending and descending aorta,^32^ in our murine study we analysed the descending aorta and thus cannot exclude the possibility of a *Phactr1*-mediated effect on vascular function in the ascending aorta. In our study, we observed no difference in blood or pulse pressure across the genotypes indicating that the changes in arterial stiffness observed were unlikely to be due to genotype-specific differences in blood pressure. However, previous studies have shown a key role of *PHACTR1* in stress fibre assembly and cellular motility.^33^ Indeed, *PHACTR1* is expressed not only in endothelial cells and monocytes but also in vascular smooth muscle cells.^34^ Thus *PHACTR1*-mediated changes in the cytoskeletal network may account for the changes in arterial stiffness observed in the current study. Future studies investigating arterial distensibility in arteries from multiple vascular beds in the *Phactr1*^−/−^ mice will be key to understanding the mechanism of *Phactr1*-mediated changes in distensibility.

The AA allele at the rs9349379 locus is associated with SCAD. We investigated if changes in distensibility were observed in patients who had previously had a SCAD. Overall, as with the ascending aorta, we observed that the CAD allele GG was associated with reduced distensibility compared with the SCAD allele. However, when this population was subdivided this observation was driven by differences in matched HV with the association no longer significant in patients who had a SCAD. Very little is known regarding the mechanism which precedes dissection of the coronary artery and how susceptibility to SCAD impacts on the function of remote arteries. Genetic studies have shown an association of SCAD with conditions linked to abnormalities in connective tissue including Marfan, Loeys Dietz, and adult polycystic kidney disease.^35,36^ This links with our current data which support a role for *PHACTR1* in structural vascular changes. Future studies should investigate how loss and gain of function of *PHACTR1* impacts vascular smooth muscle cell stiffness, extracellular cell matrix generation, and cell–cell and cell–matrix adhesion.

### 4.1 Study limitations

SCAD is a relatively rare event limiting the number of patients in this study. A larger cohort would enable a more detailed analysis of the pressure distensibility relationship in these patients. Multiple studies have shown that the rs9349379 locus is associated with changes in *PHACTR1* expression which strongly implicates *PHACTR1* as the causal gene at this locus.^4,12^ However, a previous study has also implicated endothelin-1 at this locus.^11^ Endothelin-1 is associated with both vasodilation and reduced blood pressure via its action on the ET_B_ receptor on endothelial cells and vasoconstriction and hypertension via its action on ET_A&B_ receptors on vascular smooth muscle cells. We did not find any impact of genotype on blood pressure in our patient population and there were no differences in clinical measures of vasomotor function. However, our focus here was on arterial vasodilation rather than vasoconstriction. Further studies may be needed to definitively rule out Endothelin-1 as a mediator of the *PHCTR1 locus*. As expected in a clinical population with advanced CAD we observed a high degree of variability in measures of vascular function, thus we cannot exclude the possibility that small genotype effects may exist in this population.

## 5. Conclusion

In conclusion, we have shown a role for *PHACTR1* in arterial compliance across multiple vascular beds. Interestingly, this association was not observed in SCAD patients. Further research will be key to understanding if this loss of association is causal. Our study suggests that the role of *PHACTR1* within the vasculature is primarily structural, with a minimal role for *PHACTR1* in dynamic changes in vascular tone. Future studies investigating the role of *PHACTR1* in vascular smooth muscle cell stiffness and extra cellular matrix and how this is altered in SCAD would help to address the mechanism by which *PHACTR1* mediates changes in vascular compliance.

## Supplementary material

Supplementary material is available at Cardiovascular Research online.

## Supplementary Material

cvac092_Supplementary_Data

## Data Availability

The data underlying this article will be shared on reasonable request to the corresponding author.
