# Supplementary material for: PHACTR1 modulates vascular compliance but not endothelial function: a translational study
Source: Cardiovasc Res. 2022 Jun 2;119(2):599–610. doi: 10.1093/cvr/cvac092 (PMC10064844; doi:10.1093/cvr/cvac092)
Supplement: cvac092_Supplementary_Data [file cvac092_supplementary_data.docx]

***PHACTR1* modulates vascular compliance but not endothelial function: a translational study**

Alice Wood^#^, Alexios Antonopoulos^#^, Surawee Chuaiphichai, Theodosios Kyriakou_,_ Rebeca Diaz, Abtehale Al Hussaini, Anna-Marie Marsh, Manjit Sian, Mitul Meisuria, Gerry McCann, Victoria S. Rashbrook, Edward Drydale, Sally Draycott, Murray David Polkinghorne, Ioannis Akoumianakis, Charalambos Antoniades, Hugh Watkins_,_ and *Keith M. Channon, David Adlam and *Gillian Douglas

^#^ = Both authors contributed equally

^$^ = Authors jointly supervised this work

**Supplementary Material**

**Supplementary Table 1: Demographics for CABG patients**

|  | AA | GA | AA |
| --- | --- | --- | --- |
| Age (mean±SD) | 66.6±9.8 | 66.5±9.4 | 66.4±10.4 |
| BMI, Kg/m^2^ (mean±SD) | 29.1±5.5 | 28.3±4.3 | 28.7±4.2 |
| Male sex (%) | 84.9% | 87.4% | 83.3% |
| Smoking (%) | 49.8% | 53.8% | 55.8% |
| Diabetes (%) | 18.5% | 23.1% | 26.1% |
| Dyslipidaemia | 83.7% | 83.6% | 77.5% |

**Supplementary Table 2: Demographics for SCAD and Healthy volunteers**

|  | SCAD | HV |
| --- | --- | --- |
| Age (mean±SD) | 44.1 ±8.4 | 41.8±7.3 |
| BMI, Kg/m^2^ (mean±SD) | 25.3 ±5.0 | 26.2±6.2 |
| Female Sex (%) | 98% | 95% |
| Smokers | 36% | 28% |
| Diabetes | 0% | 0% |
| Hypertension | 18% | 0% |
| Dyslipidaemia | 8% | 0% |
| Pulse pressure (mmHg, mean±SD) | AA=47±11, AG=47±12, GG=58±23 | AA=51±12, AG=51±14, GG=49±11 |
| Systolic Blood pressure (mmHg, mean±SD) | AA=118±15, AG=123±16, GG=121±16 | AA=124±14, AG=132±14, GG=122±10 |
| Diastolic Blood pressure (mmHg, mean±SD) | AA=71±12, AG=75±13, GG=69±13 | AA=72±9, AG=81±10, GG=73±8 |
